# The Immune Factors Involved in the Pathogenesis, Diagnosis, and Treatment of Sjogren's Syndrome

**DOI:** 10.1155/2013/160491

**Published:** 2013-07-09

**Authors:** Yi-fan Huang, Qian Cheng, Chun-miao Jiang, Shu An, Lan Xiao, Yong-chao Gou, Wen-jing Yu, Lei Lei, Qian-ming Chen, Yating Wang, Jun Wang

**Affiliations:** State Key Laboratory of Oral Diseases, West China Hospital of Stomatology, Sichuan University, Chengdu 610041, China

## Abstract

Sjogren's syndrome (SS) is a systemic, autoimmune disorder characterized by salivary insufficiency and lymphocytic infiltration of the exocrine glands. Even though the mechanism of its pathology and progression has been researched ever since its discovery, the roles of different parts of immune system remain inconclusive. There is no straightforward and simple theory for the pathogenesis and diagnosis of Sjogren's syndrome because of the multiple kinds and functions of autoantibodies, changing proportion of different T-lymphocyte subsets with the progression of disease, unsuspected abilities of B lymphocytes discovered recently, crosstalk between cytokines connecting the factors mentioned previously, and genetic predisposition that contributes to the initiation of this disease. On the other hand, the number of significant reports and open-label studies of B-cell depletion therapy showing clinical efficacy in sjogren's syndrome has continued to accumulate, which provides a promising future for the patients. In a word, further elucidation of the role of different components of the immune system will open avenues for better diagnosis and treatment of SS, whose current management is still mainly supportive.

## 1. Introduction

Xerostomia is a common and primary symptom during clinical practice, which could be caused by various factors, mainly Sjogren's syndrome. Sjogren's syndrome (SS) is an autoimmune disease with the hallmark of clinical features of salivary insufficiency and pathological features of focal, periductal, and perivenular lymphocytic infiltrates. The association between dry eyes and dry mouth was first noticed by Hadden in 1888, who introduced the term xerostomia [1]. In 1933, Dr. Henrik Sjogren published the most comprehensive article on this subject describing a cluster of women in a small Swedish town presenting with keratoconjunctivitis, lymphoid infiltrations of the conjunctiva, cornea, lacrimal glands, and parotid glands, a history of arthritis, swelling of the salivary glands, and dryness of the oronasopharynx. Two years later, this series of observation was connected with Mikulicz's disease and together formed the general basis for this syndrome [2]. In 1936, Duke Elder honored Sjogren by naming the disease Sjogren's syndrome. Sjogren's syndrome can be classified into two forms: primary Sjogren's syndrome characterized by keratoconjunctivitis and xerostomia without an associated autoimmune disease and secondary Sjogren's syndrome characterized by keratoconjunctivitis and xerostomia associated with an autoimmune disorder, for example, rheumatoid arthritis, systemic lupus erythematosus, and progressive systemic sclerosis. The immunological mechanism of this disease has long been studied and still been an active subject for investigation. In this review, we will discuss the function of different components of the immune system involved in the pathogenesis, progression, and treatment of this disease.

## 2. The Autoantibodies

A large number of autoantibodies have been detected in the serum of patients with Sjogren's syndrome. According to Tzioufas et al., these antibodies possess three different abilities: serving as disease markers; indicating the association with other autoimmune diseases; and exhibiting possible pathogenetic role [3].

### 2.1. Anti-Ro/SSA and Anti-La/SSB

Anti-Ro/SSA and anti-La/SSB, antibodies directed against Ro/La ribonucleoprotein complexes, can serve as a diagnostic hallmark of Sjogren's syndrome. Depending on the method applied for their identification, anti-Ro/SSA and anti-La/SSB antibodies are detected in approximately 50 to 70% of pSS patients [4]. Interestingly, anti-Ro/SSA antibodies may be found either solely or concomitantly with anti-La/SSB antibodies, whereas exclusive anti-La/SSB positivity is rare [5].

The resources of these antibodies remain sophisticated even with improved identification methods. Using a novel technique, Tengner et al. have demonstrated the presence of Ro and La autoantibody producing cells in salivary gland biopsy tissues from patients with SS [6]. And previous studies have demonstrated that anti-Ro/SSA and anti-La/SSB autoantibodies are enriched in saliva of pSS patients. It seems that the affected salivary glands are the major site of autoantibody production. These findings indicate that anti-Ro/SSA and anti-La/SSB autoantibodies are produced and assembled at sites of inflammation and imply their potential involvement in the autoimmune exocrinopathy of this disease. However, Hammi and his colleagues considered that the leakage of anti-Ro/SSA and anti-La/SSB antibodies from blood into saliva could be the main resource of autoantibodies, with the evidence that serum was shown to be significantly more sensitive than parotid saliva for the detection of Ro and La antibodies [7]. 

As a hallmark of sjogren's syndrome, it seems that the level of Ro/La antibody should remain unaltered during the subsequent course of the disease. A long-term follow-up study in the North East England by Davidson and his colleagues claimed that the serological pattern of the majority of patients remained constant throughout the follow-up period [8]. However, Fauchais et al. indicated that in a cohort of 445 pSS patients, anti-Ro/SSA and anti-La/SSB antibodies were not present at baseline but emerged secondarily in 8% of these cases, while in several cases the antibodies disappeared during followup. Some patients may exhibit pSS systemic complication during followup despite the disappearance of both hypergammaglobulinemia and anti-SSA antibodies. It is suggested that patients suspected to suffer from pSS, negative for anti-Ro/La antibodies though, should be referred for salivary gland biopsy [9].

### 2.2. Antibodies Indicating the Association with Other Autoimmune Diseases

The presence of more than one autoimmune disease in the same patient is common in clinical studies, and earlier investigations have discovered some commonalities among autoimmune diseases. For instance, as reported by Selmi et al., primary biliary cirrhosis may be considered a Sjogren's syndrome of the liver, whereas Sjogren's syndrome can be equally discussed as primary biliary cirrhosis of the salivary glands [10]. The existence of autoantibodies like rheumatoid factors and cryoglobulins, which correlate with other autoimmune diseases, may indicate the occurrence of polyautoimmunity in the prognosis of the patient.  Table 1 shows the antibodies indicating the association with other autoimmune diseases [3].

### 2.3. Anti-Muscarinic Receptor Antibodies

Whether salivary glands in patients with SS produce less saliva because of their functional or structural disturbance, induced by anti-M3R antibodies, has been a hot spot for research in recent years. Muscarinic receptors (MRs) are acetylcholine receptors coupled to G-proteins; they can be detected in plasma membranes of certain neuronal and nonneuronal cells. How the anti-M3R antibodies influence the salivary glands and which part of the receptor plays the crucial role are still controversial. A recent study by Passafaro et al. showed that anti-M3R antibodies act as a partial muscarinic agonist, which increase prostaglandin E2 (PGE2) and cyclic AMP production through modifying Na^+^/K^+^-ATPase activity and also interfere with the secretory effect of the parasympathetic neurotransmitter [11]. Using muscle strip and whole organ functional assays, Smith and Dawson showed that IgG with anti-M3R activity from patients with SS disrupted neurotransmission in tissues throughout the mouse gastrointestinal tract [12]. Several researchers have selected the second extracellular loop as the crucial part involved in the progression of pSS since it is an epitope of anti-receptor antibodies for many G-protein coupled receptors in other autoimmune conditions. Another recent study by Tsuboi et al. proved that autoantibodies against the second extracellular loop of M3R could be involved in salivary dysfunction in patients with SS by generating two hybridomas producing anti-M3R monoclonal antibodies against the second extracellular loop of M3R (anti-M3R2nd mAbs) and analyzing their function by Ca^2+^-influx assays, using a human salivary gland (HSG) cell line [13].

## 3. T Cells

It was noted in 1983 that T cells constituted the majority (>75%) of lymphocytes infiltrating the salivary glands and that CD4 T cells constituted the majority of these cells [14].

### 3.1. Th1 and Th2 Cells

Traditional opinion about these two Th subsets is that there is a dynamic balance between the two families of cytokines; while uncontrolled Th1 cells determine autoimmune states, unusual deviation to Th2 cells leads to allergic disorders [15]. The observation of Fox and Kang that increased levels of IL-1b, IL-6, tumor necrosis factor (TNF)-*α*, and IFN-*γ* have been reported in saliva from patients with SS in comparison with controls with histologically normal salivary glands, suggests that Th1 responses predominate in SS autoimmune lesions [16]. In some autoimmune cases, however, reduction of the Th1 response or a Th2 type shift may alleviate disease; many apparent exceptions are now well documented. And Th2-derived cytokines such as IL-10 have been found to be elevated in the saliva of Sjogren's syndrome patients and correlate with the severity of the disease [17]. The results of Mitsias and his colleagues reported that Type II microenvironment prevails in low-grade infiltration, while type I pattern increases in patients with definite SS, and patients with advanced lymphocytic infiltration may suggest that the cytokine pattern may shift from Th1 to Th2 as the immunopathological lesions progress [18]. Detailed mechanism of how steroid hormone and cytokines regulate the Th1/Th2 is a complex issue that needs more research, and, hopefully, some new treatment options may be drawn from this.

### 3.2. Th17 and Tregs

In spite of the role of Th1 and Th2 cells in sjogren's syndrome which remains contradictory, several data show that some abnormalities that first ascribed to Th1 cells were instead engendered by Th17 cells [19]. Th17 cells produce a family of cytokines from IL-17A through IL-17F, and, to a lesser extent, TNF-*α* and IL-22, which have been detected in the serum and saliva of SS patients, and IL-17 expression correlated with the severity of lesions. On the other hand, Tregs are thought to exert a countervailing suppressive effect on Th17 mediated cellular immunity. The reports are contradictory in that the blood of SS patients contains too many or too few Treg cells. Nguyen et al. [20] and Cornec et al. [21] found that the Th17/IL-23 system is upregulated in C57BL/6.NOD-Aec1Aec2 mice and SS patients at the time of disease and proposed the hypothesis that in the presence of IL-6, the T-cell differentiation might switch from Treg-cell pathway to Th17 pathway. As well as rheumatoid and systemic lupus erythematosus, shown by earlier investigations, IL-6 has been detected in the salivary glands of patients with SS, which not only participates in the generation of Th17 but also fosters their proliferation. IL-18, TGF-*β*, and other cytokines participate in the regulation of T cells in a complicated way, and more investigations are needed to be clarified.

## 4. B Cells

Although T cells were originally considered to play the initiating role in the autoimmune process, while B cells were restricted to autoantibody production, recent years have seen growing evidence that the roles of B cells in pSS pathophysiology are multiple and that these cells may actually play a central role in the development of the disease [20, 21].

Hansen and his colleagues reveal that patients with pSS show a general reduction of their CD27+ memory B-cell levels in peripheral blood, while an accumulation/retention of these cells is observed in their salivary glands [22, 23]. It has been reported in previous studies that the abnormal differentiation of B cells in pSS leads to a decline in circulating memory B cells and a subsequent increase in levels of plasma cells and long-lived plasma cells. In a word, the distribution and function of B cells have been reported to be altered in pSS. Aqrawi et al. observed similar phenomenons and took progress in confirming that the decrease in memory B-cell levels in the peripheral blood of patients with pSS is specific to anti-Ro and anti-La memory B cells, while whether the increase in memory B-cell infiltrates in salivary glands is also caused by anti-Ro- and anti-La-specific memory B cells needs further studies [24].

The respective membrane expression of IgD and CD38 distributes mature B cells (Bm) into sequential stages from Bm1 through Bm5 cells. These subsets can also be detected in peripheral blood, suggesting that a part of the cells maturating in secondary lymphoid organ can escape from GC and rejoin circulation. B-cell subset distribution is altered in the blood of patients with pSS, with an increase in Bm2/Bm2′ and a decrease in eBm5/Bm5. The ratio (Bm2/Bm2′)/(eBm5/Bm5) could even be considered as a diagnostic tool for the pSS [25].

Despite producing autoantibodies and cytokines, B lymphocytes can induce epithelial cells of salivary glands into apoptosis in sjogren's syndrome. By using coculture experiments with human salivary gland (HSG) cell line cells and tonsilar B lymphocytes, Varin et al. observed that direct HSG-cell-B-lymphocyte contacts were able to induce apoptosis in epithelial cells. This B-lymphocyte-mediated cell death could not be ascribed to Fas-FasL ligand interactions but required translocation of protein kinase C delta (PKC *δ*) into the nucleus of epithelial cells [26].

### 4.1. BAFF

BAFF (for B-cell activating factor of the TNF family), which is produced by all sorts of macrophages and DCs and from epithelial cells and activated T lymphocytes, has been demonstrated to be key in B-cell homeostasis. BAFF is critical for B cells to survive in the periphery and is also involved in B-cell selection by dictating set points for mature primary B-cell numbers and adjusting thresholds for specificity-based selection during downstream differentiation. As shown by many researchers, Daridon et al., for example, BAFF level is increased in the saliva of the patients and may correlate with the severity of pSS [27]. In addition, BAFF transgenic mice manifest T2-cell hyperplasia in their exocrine glands, as reported by Groom et al., and developed systemic lupus erythematosus and SS-like disease. However, there are also reports about the serum levels of BAFF that remain within, or even below, normal levels in a proportion of SS patients, indicating that the BAFF levels may fluctuate with inflammatory activity, the variations of the detection assays, and the progression of pSS [28]. Since this cytokine is crucial at several levels in the pathogenesis of pSS, it could serve as an ideal target for treatment, and precise examination methods will be needed. Two different classes of BAFF antagonists are at various stages of clinical development, selective BAFF blockers (anti-BLyS, belimumab, or Lymphostat B), and nonselective BAFF blockers (human Ig Fc), and given the importance of BAFF, further clinical trials are needed for its use in human.

### 4.2. B-Cell Depletion Therapy

Overexpression of soluble factors, such as antibodies, IFN-*α*, and BAFF, as described previously, plays an important role in the initiation and continuation of pSS. Depletion of circulating B cells may provide an opportunity to reset the immune tolerance and regulate the abnormal functions of B cells, as well as, if possible, influence the T-cell compartment, since B cells participate in the antigen prestation and produce several important cytokines in the differentiation of T cells. The most widely studied target for achieving B-cell depletion is the CD20 antigen, a transmembrane protein found on pre-B and mature B cells but not on pro-B cells and normal plasma cells. The only anti-CD20 biologic agent tested in pSS is rituximab. Although the direct pathophysiological role of B cells in glandular tissue destruction in pSS has not been fully elucidated, B-cell depletion therapy with rituximab appears to be successful and improves salivary flow, which was reported by Meijer and his groups in 2010 [29, 30]. Meiners et al. reviewed 20 reported trials evaluating the effect of rituximab in pSS patients and found great discrepancy among the results. It seems that the result we can conclude from these trials is that pSS patients with early, active disease with extraglandular manifestations are most likely to benefit the most from rituximab treatment [31].

As detailed before, the cytokine BAFF plays a key role in B-cell differentiation, survival, and activation. As reported by Pers and his colleagues, following placebo-controlled RTX trial in pSS after B-cell depletion therapy with RTX showed an increase of BAFF in serum levels, indicating the role of BAFF in the repopulation of B cells after treatment [32]. In conclusion, targeting BAFF in combination with B-cell depletion seems logical and may hold therapeutic promise for pSS.

## 5. Immunogenetics of pSS

Immunogenetic mechanism of pSS is another issue that has been heatedly investigated, using a different kind of mouse models, including MRL/lpr, NZB/NZW F1-hybrids, nonobese diabetic (NOD), NFS/sld, and several new strains like Id3 gene knock-out (KO) mouse, the aromatase gene KO mouse, the Baff gene knock-in mouse, and the IQI/Jic mouse and the C57BL/6.NOD-Aec1Aec2 congenic line. Not surprisingly, studies using animal models of sjogren's syndrome indicate that disease susceptibility is multigenic, encompassing many critical causal elements [2].

Observations reported by Harley et al. suggest that pSS susceptibility is linked to HLA-DQ genes, specifically DQ1 and DQ2, when associated with the presence of anti-SS-A/Ro and anti-SS-B/La autoantibodies [33]. A study by Tzioufas using sera from 90 pSS patients from different countries in Europe showed a significant association of anti-Ro/SSA anti-La/SSB with the presence of HLA-DRB1∗03 and DQB1∗02, while the strength of this association varied with countries in which the data was collected. However, different MHC genetic linkages should be expected in different ethnic groups. The discrepancy among the results from different countries may be merely because of the relative homogeneity of the populations studied [34, 35].

Since abnormalities of the NF-*κ*B signal transduction pathway and phosphorylation of I*κ*B*α* in several autoimmune diseases are observed, analysis of the gene and protein expression profiles of SS monocytes has been carried out by Lisi et al. Their findings clearly demonstrate changes in the levels of I*κ*B*α* in SS monocytes, suggesting that the attenuated expression of I*κ*B*α* could contribute to the deregulation of NF-*κ*B pathways in the SS pathogenesis. Decreased expression of I*κ*B*α* may specifically amplify cytokines production and inflammatory response linked to Sjogren's syndrome [36].

Polymorphisms in genes encoding cytokines or factors implicated in cytokine signaling are also thought to participate in disease pathogenesis. As shown by Cay and his colleagues, polymorphism in the TNF-alpha gene promoter at position-1031 is associated with increased circulating levels of TNF-*α*, myeloperoxidase, and nitrotyrosine in primary Sjogren's syndrome [37].

## 6. Conclusion 

Sjogren's syndrome is a multifaceted autoimmune disease. Even though the mechanism of its pathology has been studied since its discovery, the roles of different populations of immune cells remain inconclusive. How the cells and cytokines interact to promote the development of SS is a highly promising field of investigation. With the development of techniques, the complicated underlying genetic factors involved in pSS are promising to be identified. Although investigations about treatments targeting the immune factors participating in the progression of pSS show some positive outcome, more clinical trials are required before their application in human.

## Figures and Tables

**Table 1:** Prevalence and associated diseases of autoantibodies.

Antibodies	Prevalence (%)	Related autoimmune diseases
Rheumatoid factors (RF)	40–50	Hypergammaglobulinemia
Cryoglobulins	10–15	Lymphoma development and death
Anti-centromere antibodies (ACA)	4–17	Systemic sclerosis
Anti-mitochondrial antibodies (AMA)	5–6.5	Primary biliary cirrhosis
Anti-cyclic citrullinated peptide antibodies (anti-CCP)	7–10	Articular manifestations
Anti-smooth muscle antibodies (ASMA)	6.5–62	Autoimmune hepatitis
